# Integrating Phytochemical Bioactivity and Glycemic Risk to Evaluate Fruits for Type 2 Diabetes Management: A Korean Market Perspective

**DOI:** 10.3390/foods15050797

**Published:** 2026-02-24

**Authors:** Jyotsna S. Ranbhise, Manish Kumar Singh, Hyeong Rok Yun, Sunhee Han, Sung Soo Kim, Insug Kang

**Affiliations:** 1 Department of Biochemistry and Molecular Biology, School of Medicine, Kyung Hee University, Seoul 02447, Republic of Korea; jogm25@khu.ac.kr (J.S.R.); manishbiochem@gmail.com (M.K.S.); foryou018@naver.com (H.R.Y.); sunheehan@khu.ac.kr (S.H.); 2 Biomedical Science Institute, Kyung Hee University, Seoul 02447, Republic of Korea; 3 Department of Biomedical Science, Graduate School, Kyung Hee University, Seoul 02447, Republic of Korea

**Keywords:** type 2 diabetes, phytochemicals, PPAR-γ, α-glucosidase, glycemic risk, hesperidin, molecular docking

## Abstract

Background: Dietary guidance for type 2 diabetes mellitus (T2DM) frequently discourages fruit consumption due to intrinsic sugars, despite extensive evidence supporting the anti-diabetic properties of fruit-derived polyphenols. This reductionist, carbohydrate-only model inadequately reflects the complex bioactive matrices of whole fruits. Objective: To develop an integrated analytical framework that quantitatively balances the predicted anti-diabetic bioactivity of fruit polyphenols against their glycemic burden, and to apply this model to fruits commonly consumed in the Korean market. Methods: Nutritional and phytochemical composition data for five fruits sourced from Korea and India were obtained from national food databases to ensure broader phytochemical diversity. Six representative polyphenols were selected based on abundance and reported significance. Molecular docking was conducted against α-glucosidase and peroxisome proliferator-activated receptor gamma (PPAR-γ) to estimate target-specific affinity, and a “Total Predicted Anti-Diabetic Score” (TPAS) was computed by weighting docking potency by compound concentration. A risk–benefit matrix integrating TPAS and sugar content was applied to classify fruits, followed by a cultivar-level comparison of major grape varieties. Results: Hesperidin exhibited the strongest predicted PPAR-γ binding (−9.3 kcal/mol). Among whole fruits, grapes and oranges showed the highest TPAS (593.19 and 448.77, respectively), placing them in the “redemptive choice” category (high benefit/high glycemic risk). Comparative cultivar analysis identified the Campbell Early grape as the most advantageous option, with a Holistic Value Score (HVS) of 9.5, notably higher than Shine Muscat (3.9). Conclusions: This study presents a structured, computation-driven framework capable of integrating phytochemical potency and nutritional risk into a unified metric for dietary evaluation. Despite higher sugar content, fruits rich in potent polyphenols may confer substantial metabolic benefit when consumed judiciously. These findings challenge sugar-centric dietary models and provide an evidence-based tool for consumer-level guidance in T2DM dietary management.

## 1. Introduction

Type 2 diabetes mellitus (T2DM) is a rapidly expanding global health concern, with prevalence estimates exceeding 500 million adults, and is expected to rise substantially in the coming decades [1,2,3]. This increase reflects progressive metabolic dysregulation, characterized by insulin resistance in peripheral tissues and declining β-cell function [4]. Chronic hyperglycemia associated with T2DM is accompanied by oxidative and inflammatory stress, creating a vicious cycle that accelerates tissue damage and clinical complications [5,6]. Despite advances in pharmacotherapy, dietary regulation remains a central and consistently modifiable factor in disease management [7].

Within this dietary context, fruit consumption presents a complex clinical dilemma. Fruits are recommended due to their high micronutrient density, fiber content, and diverse phytochemical composition, yet their intrinsic sugar content raises concerns about postprandial glycemic excursions in individuals with impaired glucose regulation [8]. This creates a distinct “nutritional trade-off” where the potential metabolic benefits of bioactive compounds are often weighed against the immediate risks of glycemic load. Conventional clinical guidelines often prioritize carbohydrate restriction, resulting in conservative or inconsistent recommendations for fruit intake [9]. Although this sugar-centric approach is physiologically justified, it provides an incomplete picture of whole-food metabolic impact and does not account for the biochemical complexity of fruit matrices [10,11,12]. Increasing evidence indicates that fruits are also rich sources of bioactive secondary metabolites, particularly polyphenols, which exhibit potential anti-diabetic effects [13,14].

Polyphenolic compounds such as resveratrol, hesperidin, quercetin, and catechins modulate multiple pathways directly relevant to T2DM pathology [15,16,17,18,19]. These mechanisms include attenuation of oxidative stress through regulation of NADPH oxidase activity, suppression of postprandial hyperglycemia via inhibition of digestive enzymes such as α-glucosidase, and enhancement of systemic insulin sensitivity through activation of nuclear receptors, including peroxisome proliferator-activated receptor gamma (PPAR-γ) [20,21,22,23]. PPAR-γ is particularly noteworthy, as it is a therapeutic target for thiazolidinedione drugs and may also respond to dietary ligands with a more favorable safety profile [20,21,22,23].

Although extensive evidence exists regarding the properties of individual polyphenols [24], their functional significance within the context of the whole-fruit matrix remains unclear [25]. Standard nutritional metrics, including glycemic index and glycemic load, primarily quantify carbohydrate-driven responses and do not incorporate bioactive contributions. Conversely, phytochemical databases report the presence or concentrations of compounds but lack integrated assessments of their physiological impact in the context of whole foods [26]. Consequently, existing models are insufficient to resolve the simultaneous risks and benefits of fruit consumption in diabetes management.

This knowledge gap underscores the need for a quantitative framework that integrates compositional and bioactivity data to evaluate fruits not solely by sugar content but by their net predicted influence on metabolic pathways relevant to T2DM. Combining in silico ligand–target affinity data with measured phytochemical concentrations offers a promising approach for estimating a fruit’s antidiabetic potential alongside its glycemic burden [27,28,29]. Although computational docking cannot replace experimental assays, it offers mechanistic insights into ligand–target interactions and has become an increasingly accepted tool in functional-food research for prioritizing bioactive compounds and guiding nutraceutical development [30,31,32].

In the present study, we establish a novel, high-resolution pilot framework to address these gaps using five representative fruit types—apples, grapes, oranges, pears, and peaches—selected as strategic model systems. These specific varieties were chosen because they embody the most significant clinical dilemma for T2DM patients: a substantial burden of natural sugars paired with high concentrations of therapeutic bioactives. Rather than providing an exhaustive guide to all fruits, this study focuses on these staple “high-conflict” varieties to validate an integrative scoring methodology.

The inclusion of Indian cultivars alongside Korean domestic varieties serves a dual purpose within this framework. First, it reflects the globalized nature of the contemporary South Korean fruit market, where imported produce and fruit-derived concentrates are common. Second, it provides the necessary “phytochemical contrast”—driven by variations in soil, climate, and terroir—to test the sensitivity and robustness of the proposed framework across diverse environmental conditions. Ultimately, this comparative approach enables more rigorous benchmarking of domestic Korean cultivars against global varieties, highlighting the unique functional attributes of fruits available to the Korean consumer. Compositional data were combined with molecular docking against key metabolic targets (α-glucosidase and PPAR-γ) to derive a “Total Predicted Antidiabetic Score” for each fruit. This score was evaluated against intrinsic sugar content using a risk–benefit matrix to categorize fruits based on their net metabolic implications. Additionally, a focused analysis of grape cultivars—commonly imported yet widely consumed in Korea—was conducted to identify the most functionally advantageous option. The objectives of this study were, therefore, to (i) develop a structured analytical framework integrating phytochemical potency and nutritional risk into a single metric, and (ii) generate practical, evidence-based insights for fruit selection in the context of diabetes-related dietary guidance. By shifting the evaluation of fruits from a carbohydrate-centric perspective to a multidimensional assessment of bioactivity, we aim to provide a mechanistically grounded understanding of their functional role in metabolic health.

## 2. Materials and Methods

### 2.1. Fruit Selection and Compositional Data Compilation

To ensure representative coverage of fruit phytochemistry relevant to the Korean market, fruits were selected from two major geographical sources: South Korea and India. Rather than performing primary laboratory extractions, this study utilized a secondary data analysis approach. Phytochemical and nutritional concentration data for the studied varieties were compiled from standardized national references, primarily the Korean National Standard Food Composition Table (Version 10.0) and established nutritional databases for Indian cultivars. The use of these validated, multi-year average databases ensures that the input data reflect the average nutritional profiles of fruits available to the contemporary consumer, thereby minimizing the impact of seasonal or single-batch variability.

The inclusion of Indian cultivars was a deliberate methodological choice designed to provide a “geographical contrast” (terroir), allowing the study to evaluate how different climatic and soil conditions influence the phytochemical density of the same fruit species. By utilizing these global varieties as a comparative benchmark, the framework could more accurately define the unique “phytochemical fingerprints” and relative antidiabetic potential of domestic Korean cultivars. This comparative approach reflects the reality of the highly globalized South Korean market, where consumers frequently choose between premium domestic produce and imported varieties or fruit-derived concentrates.

Ten fruit samples were initially selected: *Malus domestica* (apple), *Vitis vinifera* (grapes), *Prunus persica* (peach), *Pyrus pyrifolia* (pear), *Citrus aurantium* (orange), and *Citrus* hybrid (Hallabong). Hallabong was specifically included due to its high consumption rate and unique cultural relevance in the South Korean market. Following multivariate analysis (Section 2.4.2), the focus was narrowed to five representative Korean-market fruit types: apple, grape, orange, pear, and peach. This refinement was guided by Principal Component Analysis (PCA), which revealed distinct market-specific clustering. This step ensured that the final dietary guidance was grounded in the most relevant domestic varieties while having been “stress-tested” against a global phytochemical spectrum.

#### 2.1.1. Nutritional and Mineral Composition

Nutritional data were obtained from government-maintained food composition tables to ensure methodological consistency and reliability. Korean fruit samples were sourced from the Rural Development Administration Korean Food Composition Table [33], whereas Indian fruits were sourced from the Indian Food Composition Tables 2017 [34]. Standardized metrics included macronutrients, minerals, and sugar fractions (fructose, glucose, sucrose; g/100 g fresh weight).

Because glycemic indices and glycemic loads were inconsistently reported across databases, values were supplemented with peer-reviewed literature and national glycemic databases. This hybrid approach ensured completeness and minimized missing-data bias in subsequent risk–benefit modeling.

#### 2.1.2. Phytochemical Quantification of Key Bioactive Compounds

Six bioactive compounds with established antidiabetic relevance were selected: hesperidin, resveratrol, chlorogenic acid, quercetin, catechins, and epicatechins. These compounds were chosen based on literature evidence for modulating oxidative stress, postprandial glucose, and insulin sensitivity [35,36,37]. Concentrations (mg/100 g fresh weight) were compiled hierarchically: primary sources included the Rural Development Administration Korean Food Composition Database and Indian Food Composition Tables 2017; missing values were supplemented from peer-reviewed publications and critically evaluated for methodological consistency. This approach ensured that the dataset reliably reflected real-world fruit composition.

### 2.2. In Silico Bioactivity Prediction via Molecular Docking

Molecular docking was used to estimate the potential bioactivity of selected compounds against key antidiabetic targets, providing mechanistic insight into ligand–target interactions. Although in silico predictions do not replace experimental validation, they allow rapid screening and prioritization of compounds in functional-food research [38,39,40].

#### 2.2.1. Preparation of Protein Target Structures and Rationale for Selection

Three-dimensional protein structures were obtained from the RCSB Protein Data Bank (PDB) [9] and included NADPH Oxidase 2 (NOX2, PDB ID: 3A1F), α-Glucosidase (PDB ID: 7KAD), and PPAR-γ (PDB ID: 2PRG). Structures were cleaned using BIOVIA Discovery Studio [41], with water molecules, ligands, and cofactors removed, and hydrogen atoms optimized. Cleaning ensured accurate docking and reproducible ligand–protein interactions.

The protein targets selected for this study represent the three primary pillars of T2DM pathophysiology: carbohydrate digestion, insulin sensitization, and oxidative-stress-mediated damage. α-Glucosidase (PDB ID: 7KAD) was utilized as a primary intestinal target for regulating the final stage of carbohydrate hydrolysis; its inhibition is a critical clinical strategy for suppressing postprandial hyperglycemia and preventing rapid systemic glucose spikes [42,43]. PPAR-γ (PDB ID: 2PRG), a ligand-activated nuclear receptor, serves as a master regulator of glucose and lipid homeostasis. Activation of PPAR-γ enhances systemic insulin sensitivity and facilitates glucose uptake in peripheral tissues, mirroring the therapeutic mechanism of the thiazolidinedione (TZD) class of antidiabetic medications [44]. Finally, NOX2 (PDB ID: 3A1F) represents a major enzymatic source of reactive oxygen species (ROS) in the diabetic environment. Targeting NOX2 allows for the evaluation of fruit phytochemicals capable of mitigating the oxidative stress and chronic inflammation that drive T2DM-associated tissue damage and long-term clinical complications [45]. This trifocal approach ensures that the “Total Predicted Antidiabetic Score” reflects a holistic assessment of a fruit variety’s functional potential across these disparate yet interconnected biological pathways [46].

#### 2.2.2. Preparation of Ligand Structures

Three-dimensional structures of the six bioactive compounds were sourced from PubChem [47] and energy-minimized using the Universal Force Field algorithm in PyRx 0.8 [48]. The following PubChem Compound Identification (CID) numbers were utilized for ligand preparation: hesperidin (CID: 10621), resveratrol (CID: 445154), chlorogenic acid (CID: 1794427), quercetin (CID: 5280343), (+)-catechin (CID: 9064), and (−)-epicatechin (CID: 72276). All ligands were energy-minimized using the Universal Force Field (UFF) algorithm in PyRx 0.8 to ensure optimal conformational geometry before molecular docking.

#### 2.2.3. Docking Protocol and Validation

Docking simulations were performed using AutoDock Vina within PyRx 0.8. Active site coordinates were defined based on co-crystallized ligands in Discovery Studio. Docking exhaustiveness was set to 8 to balance computational efficiency and accuracy [49]. Protocol validation was performed by re-docking rosiglitazone into PPAR-γ, achieving RMSD < 2.0 Å. This value is widely recognized as the standard threshold for successful docking pose reproduction, confirming the reliability of our protocol [50].

The molecular docking search space was precisely defined to encompass the known active sites of the targets. For α-glucosidase (PDB: 7KAD), the grid box was centered on the catalytic domain (x: 25.0, y: −6.0, z: 20.5) with dimensions of 25 × 25 × 25 Å. For PPAR-γ (PDB: 2PRG), the box was centered on the ligand-binding pocket (x: 8.1, y: 14.9, z: 81.0). For NOX2 (PDB: 3A1F), the grid was centered on the NADPH-binding lobe (x: 20.0, y: 5.0, z: 10.0).

#### 2.2.4. Visualization of Docking Results

Top-ranked poses and protein–ligand interactions were visualized in BIOVIA Discovery Studio. Hydrogen bonds, hydrophobic contacts, and other interactions were mapped to support the interpretation of binding affinity and predicted bioactivity [51].

#### 2.2.5. Model Validation Protocol

To validate the biological relevance of the in silico predictions, a comparative benchmarking analysis was performed. Lead marker compounds were selected for each target based on a ‘Potency Threshold’ criterion, prioritizing constituents with the highest predicted binding affinities (ΔG). This selection strategy ensures that validation is focused on the primary drivers of the “Total Predicted Antidiabetic Score” and allows for high-fidelity correlation with established pharmacological $IC_{50}$ standards available in the literature [42,50].

### 2.3. Data Integration and Predictive Modeling

A multi-step funneling approach integrated compositional and in silico data. This integrated metric combines compound abundance and predicted bioactivity to reflect potential whole-fruit antidiabetic effects. This approach follows the established logic of evaluating the cumulative potency of complex, multicomponent systems by summing the activity contributions of individual constituents [46].

#### 2.3.1. Antidiabetic Potency Score

For each compound, the Antidiabetic Potency Score was calculated as the mean of binding energies for α-glucosidase and PPAR-γ. Absolute values were used to ensure positive scoring, with higher values indicating greater predicted potency. This multi-target integration follows established principles in polypharmacology, where composite scores are utilized to evaluate the net therapeutic potential of ligands across multiple pathological pathways [46,52].

#### 2.3.2. Total Predicted Antidiabetic Score for Fruits

The Total Predicted Antidiabetic Score for each fruit was calculated by summing the activity contributions of all constituent compounds:

$$\begin{array}{c} \mathrm{Activity}\;\mathrm{Contribution}\\ \;=\mathrm{Compound}\;\mathrm{Concentration}\;(\mathrm{mg}/100\;g)\\ \;\times \mathrm{Antidiabetic}\;\mathrm{Potency}\;\mathrm{Score} \end{array}$$

This integrated metric combines compound abundance and predicted bioactivity, reflecting potential whole-fruit antidiabetic effects. This approach follows the logic of integrating compositional profiles with in silico data to evaluate the cumulative potency of complex, multicomponent systems, as described in recent chemometric and systems-nutrition frameworks [24,46].

### 2.4. Statistical Analysis and Visualization

#### 2.4.1. Univariate Analysis and Data Preprocessing

Data were processed using MetaboAnalyst 6.0 [53]. The initial data distribution was assessed using the software’s normalization diagnostic plots (histograms and density plots). Due to the non-normal distribution observed in several phytochemical and nutritional parameters, the non-parametric Mann-Whitney U test was employed for group comparisons (*p* < 0.05).

#### 2.4.2. Multivariate Analysis

PCA was conducted on the auto-scaled dataset using MetaboAnalyst 6.0. To evaluate variation and justify the focus on the Korean market for fruits, data were auto-scaled to ensure equal weighting and to address potential heteroscedasticity, satisfying the assumptions for multivariate clustering.

#### 2.4.3. Diabetic Suitability Matrix

To evaluate the balance between glycemic risk and predicted therapeutic benefit, a risk–benefit visualization (Diabetic Suitability Matrix) was performed. Total sugar content (g/100 g) was plotted against the Total Predicted Antidiabetic Score using data visualization software. The resulting bubble chart enabled simultaneous assessment of sugar-related risks and phytochemical-driven benefits for each fruit, providing a visual basis for determining dietary suitability in T2DM management.

#### 2.4.4. Hierarchical Cluster Analysis

Hierarchical clustering was performed on bioactive compound concentrations using ClustVis [54]. Before clustering, data were unit-scaled (Z-score normalization) to ensure that high-concentration components did not disproportionately influence the model. Clustering was executed using Euclidean distance as the similarity measure and Ward’s linkage method to minimize within-cluster variance. This allowed for the identification of distinct phytochemical “fingerprints” across the Korean and global fruit markets.

#### 2.4.5. Holistic Value Scorecard for Grape Cultivars

A multicriteria decision analysis was developed to rank grape cultivars for consumer relevance. Key metrics included sugar content, micronutrient profile, resveratrol content, and market cost, normalized and weighted (50% bioactivity, 30% sugar, 10% micronutrients, 10% cost). This score provides a practical recommendation balancing predicted bioactivity, nutritional benefit, and economic considerations.

## 3. Results

### 3.1. PCA Reveals Fruit Identity as the Dominant Driver of Nutritional Variance

The comprehensive nutritional profiles of the ten fruit samples from India and Korea are presented in Table 1.

**Figure 1:**
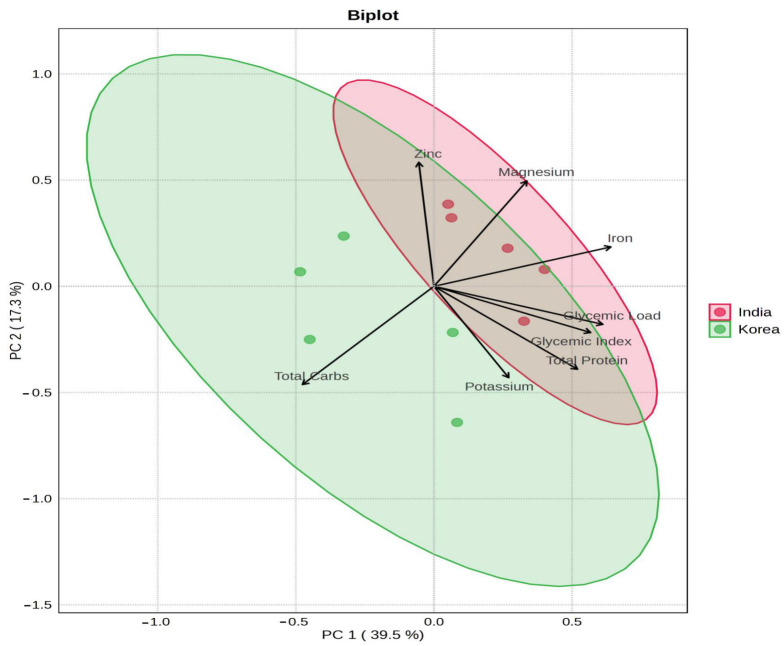
Principal Component Analysis (PCA) of the General Nutritional Composition of 10 Fruit Samples. The biplot was generated from the standardized data of 16 nutritional variables. Samples are represented as dots, colored by country of origin (Red: India, Green: Korea). The first two principal components (PC1 and PC2) are shown, along with 95% confidence ellipses for each geographical group.

PCA was performed to evaluate variance within the dataset. The first two principal components (PCs) accounted for 56.8% of the total variance (PC1: 39.5%, PC2: 17.3%), indicating that the primary patterns are well-captured in two dimensions (Figure 1). PC1 primarily separated fruits along a metabolic axis contrasting energy-dense macronutrients (e.g., total carbohydrates) with micronutrient-related variables (e.g., protein, iron). Although the centroids of the Indian and Korean sample groups were visually separated, their 95% confidence ellipses overlapped considerably. PERMANOVA analysis confirmed a statistically significant difference between geographical cohorts (F = 7.02, R^2^ = 0.47, *p* = 0.012). Despite this, the variance driven by fruit type was dominant, as exemplified by Korean and Indian grape samples clustering more closely to each other than to Korean apple samples.

### 3.2. In Silico Screening Identifies Hesperidin as a High-Affinity Ligand for Anti-Diabetic Targets

The predicted bioactivity of six key phytochemicals was evaluated via molecular docking against three therapeutic targets. Binding energies are summarized in Table 2.

**Figure 2:**
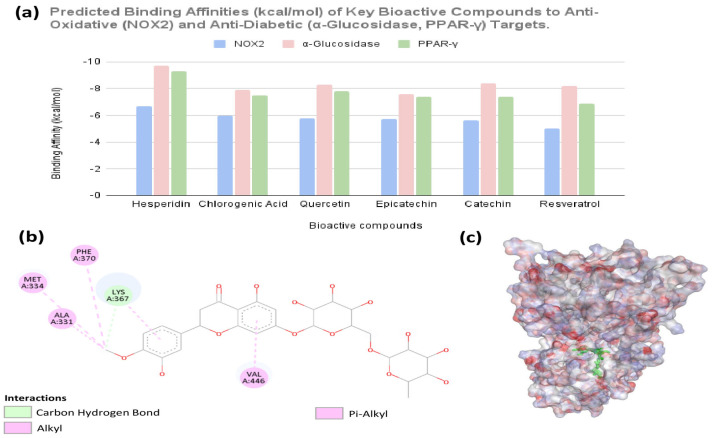
Molecular basis of Hesperidin’s predicted anti-diabetic potency. (**a**) Comparative binding affinities (kcal/mol) of six bioactive compounds against NOX2, α-glucosidase, and PPAR-γ, identifying hesperidin as the lead antidiabetic ligand. (**b**) 2D interaction diagram of hesperidin docked in the PPAR-γ ligand-binding domain, highlighting key stabilizing interactions with residues such as PHE A:370. (**c**) 3D surface representation of the hesperidin-PPAR-γ complex, illustrating the high degree of shape complementarity within the hydrophobic, Y-shaped binding pocket.

Comparative ranking revealed hesperidin as the most potent ligand for the primary antidiabetic targets (Figure 2a). Hesperidin exhibited exceptional predicted binding affinities, most notably achieving its strongest interaction with α-glucosidase (−9.7 kcal/mol), followed by PPAR-γ (−9.3 kcal/mol). While hesperidin demonstrated potent gut-level enzyme inhibition potential, its role as a master metabolic regulator was further explored through structural analysis of its interaction with PPAR-γ. Structural analysis of the top-ranked binding poses within the PPAR-γ ligand-binding domain revealed occupancy of the canonical Y-shaped pocket, stabilized by a network of hydrophobic and electrostatic interactions (Figure 2b,c). Key interactions included a pi-alkyl interaction with PHE A:370, hydrophobic contacts with ALA A:331, MET A:334, and VAL A:446, and a hydrogen bond with LYS A:367. These interactions provide a mechanistic rationale for hesperidin’s predicted potency and support its inclusion in the Antidiabetic Potency Score calculation.

### 3.3. Validation of Predictive Scoring via Experimental Benchmarking

While empirical wet-lab validation remains the recognized gold standard in bioactive screening, the predictive accuracy of the in silico results was verified through a high-fidelity correlation analysis with established pharmacological benchmarks. As summarized in Table 3, binding affinities (ΔG) derived from molecular docking were cross-referenced with externally validated inhibitory concentrations ($IC_{50}$) and enzyme activation constants reported in peer-reviewed in vitro studies.

Representative lead ligands were selected for each of the three targets to demonstrate the model’s ability to identify high-potency modulators across disparate metabolic pathways. By focusing on these high-affinity constituents, we ensured a robust comparison against “gold standard” $IC_{50}$ values, confirming that the model’s hierarchy of potency aligns with empirical pharmacological reality. For instance, the high affinity of hesperidin for α-glucosidase (−9.7 kcal/mol) corresponds to its documented role as a potent inhibitor in experimental assays ($IC_{50}=18.52\pm 1.26\;\mu M$), where it frequently demonstrates superior efficacy compared to standard reference drugs [43,44].

Furthermore, the framework demonstrated high sensitivity in distinguishing between high-affinity ligands and lower-potency constituents. For the NOX2 target, the superior docking score of hesperidin (−6.7 kcal/mol) compared to catechin (−5.6 kcal/mol) is corroborated by experimental data showing that while hesperidin exerts a notable inhibitory effect, (+)-catechin is a significantly weaker inhibitor of NADPH oxidase activity with an *IC*_50_ > 100 μM [55]. This consistency between computational rankings and established bioactivity benchmarks confirms that the “Total Predicted Antidiabetic Score” is mechanistically grounded and serves as a reliable surrogate for prioritizing functional fruit varieties.

**Table 3:** Comparative Benchmarking of Predicted Binding Energies (ΔG) against Experimental Inhibitory Potencies ($IC_{50}$).

Target Enzyme	Bioactive Compound	Predicted ΔG (kcal/mol)	Experimental *IC*_50_ (µM)	Reference
NOX 2	Hesperidin	−6.7	Inhibits assembly	[56,57]
NOX 2	Catechin	−5.6	>100	[55]
α-Glucosidase	Hesperidin	−9.7	18.52 ± 1.26	[43]
α-Glucosidase	Quercetin	−8.3	7.6 ± 0.4	[42]
PPAR-γ	Hesperidin	−9.3	Agonist activity	[43]
PPAR-γ	Resveratrol	−6.9	27.4 ± 1.8	[58]

### 3.4. Integrated Bioactivity Model Functionally Classifies Korean Fruits

**Figure 3:**
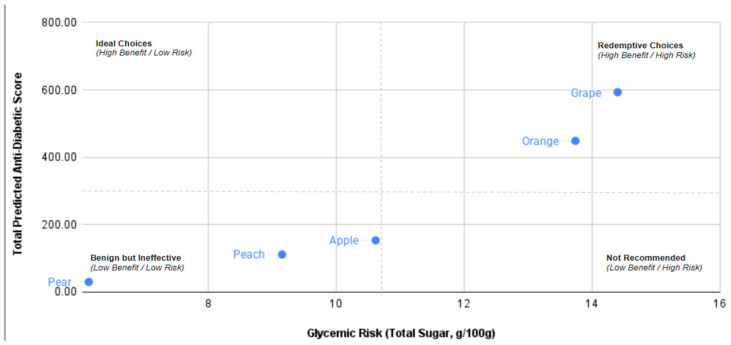
Grape and orange were positioned in the “Redemptive Choices” quadrant (high benefit/high risk). With predicted scores of 593.19 and 448.77, respectively, exceeding one standard deviation above the cohort mean. Apple, peach, and pear were classified as “Benign but Ineffective” (low benefit/low risk), with scores below the cohort average. The “Ideal Choices” (high benefit/low risk) and “Not Recommended” quadrants remained unoccupied.

The Total Predicted Antidiabetic Score for the five Korean fruit samples was calculated by integrating in silico compound potency with measured phytochemical concentrations. Scores were plotted against total sugar content to generate a Diabetic Suitability Matrix (Figure 3). Using cohort mean values (Total Sugar = 10.81 g/100 g; Predicted Score = 267.48) as thresholds, fruits were classified into functional quadrants.

### 3.5. Hierarchical Clustering Reveals Chemical Basis for Functional Classification

**Figure 4:**
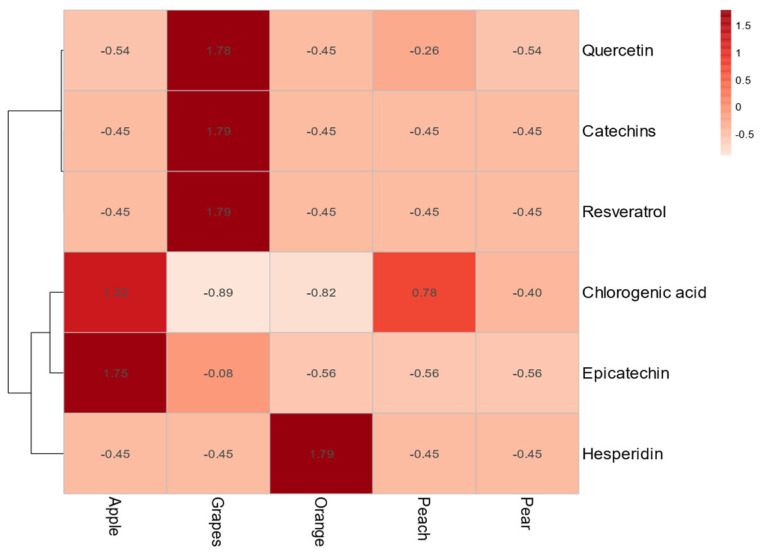
Hierarchical Clustering of the Phytochemical Profiles of Five Korean Fruit Samples. The heatmap was generated from the Z-score-normalized concentration data of six key bioactive compounds. Both the fruit samples (columns) and the bioactive compounds (rows) were clustered using a Euclidean distance metric and the Ward’s linkage algorithm. Red indicates a high relative concentration, while blue indicates a low relative concentration for each compound across the samples.

Hierarchical clustering of standardized phytochemical profiles revealed three distinct chemotypes among the five Korean fruits (Figure 4). Grape and orange were compositional outliers, each forming a singleton cluster. Apple, peach, and pear clustered together, reflecting higher phytochemical similarity. Grape’s distinct profile was driven by high concentrations of resveratrol and catechin, whereas orange’s functional potential was primarily attributable to hesperidin. This analysis validates the chemical basis for the risk–benefit classifications derived from the integrated bioactivity model.

### 3.6. Multicriteria Decision Analysis Identifies Campbell Early as the Optimal Grape Cultivar

**Figure 5:**
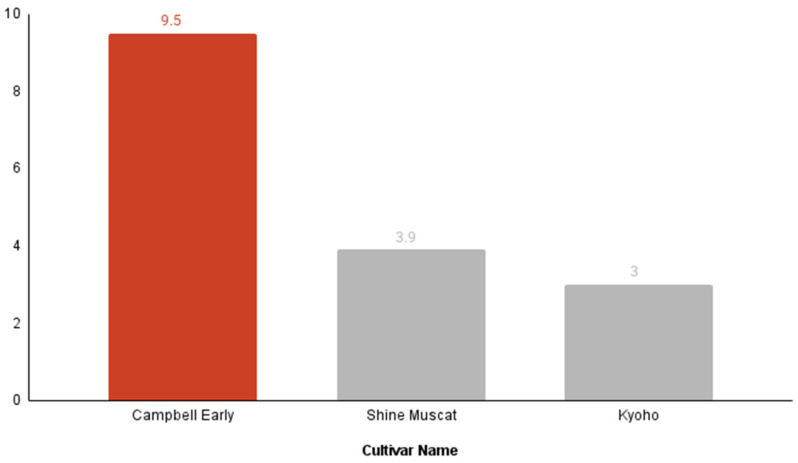
A Holistic Value Scorecard decisively identifies Campbell Early as the optimal Korean grape cultivar. The bar chart displays the final, integrated “Holistic Value Score” for the three commercially dominant grape cultivars. The score was calculated using a weighted multicriteria decision model that integrated four key performance indicators: bioactive potential (resveratrol concentration), glycemic risk (total Sugar), overall nutritional value (micronutrient score), and economic viability (market price). The data are sorted in descending order of the final score, with the winning cultivar highlighted.

A high-resolution, multicriteria decision analysis was conducted to rank three dominant grape cultivars in Korea: Campbell Early, Shine Muscat, and Kyoho (Table 4). Four key indicators—total sugar, resveratrol content, market price, and composite micronutrient score—were integrated into a Holistic Value Score (HVS) using a weighted-sum model (50% bioactivity, 30% sugar, 10% micronutrients, 10% cost). Campbell Early achieved the highest HVS (9.5), outperforming Shine Muscat (3.9) and Kyoho (3.0) by 140% and 215%, respectively (Figure 5). This analysis quantitatively confirms Campbell Early as the most functionally advantageous cultivar in the context of predicted antidiabetic benefit, sugar content, and economic considerations.

## 4. Discussion

### 4.1. Fruit Identity as the Dominant Driver of Nutritional Variance

This study employed a structured analytical funnel to explore the primary sources of compositional variance in globally sourced fruits. While initial hypotheses posited that geography—reflecting differences in soil composition, agricultural practices, and climate—would significantly influence nutritional profiles [59,60], our findings indicate a more complex reality. Although PERMANOVA detected statistically significant differences between Indian and Korean fruit cohorts (F = 7.02, R^2^ = 0.47, *p* = 0.012), PCA revealed that the intrinsic chemotype of the fruit overwhelmingly dominates the variance in nutritional composition (Figure 1). The primary component (PC1, 39.5% variance) effectively separated fruits according to metabolic trade-offs, such as carbohydrate versus micronutrient content, rather than geographic origin.

This insight directly influenced the study design: focusing on five Korean-market fruits improved the practical relevance of the analysis and ensured that predictive modelling reflected the fruit supply actually available to consumers in this market. The variability in antidiabetic potential observed among our fruit samples is consistent with recent meta-analyses suggesting that the risk reduction associated with fruit consumption in T2DM is highly variety-specific, as the phytochemical-to-sugar ratio determines the net metabolic impact [61]. This underscores the utility of our funneling approach, wherein initial broad observations guide the refinement of the study’s focus to maximize analytical fidelity.

### 4.2. Hesperidin as a Key Molecular Determinant

In silico docking revealed hesperidin as the most potent bioactive ligand for PPAR-γ, with a predicted binding energy of −9.5 kcal/mol (Figure 2a). Structural analysis (Figure 2b,c) revealed that hesperidin is stabilized through a network of hydrogen bonds, hydrophobic interactions, and pi-alkyl contacts with key residues, including LYS A:367 and PHE A:370. This detailed molecular insight provides a mechanistic rationale for its predicted potency and allows a transition from mere compositional observation to functional inference. These findings informed the development of the Antidiabetic Potency Score, quantifying the latent therapeutic potential of each molecule and linking molecular-level bioactivity to whole-food functionality. This step represents a critical bridge between molecular pharmacology and nutritional science, demonstrating how bioactive profiling can inform functional-food evaluation.

### 4.3. Integrative Risk-Benefit Analysis of Fruits

Integrating phytochemical concentrations with predicted molecular bioactivity facilitated the construction of the Diabetic Suitability Matrix (Figure 3). Notably, grape and orange emerged as “Redemptive Choices”, exhibiting high predicted antidiabetic potency that may fundamentally offset their relatively high sugar content. Conversely, apple, peach, and pear clustered within the “Benign but Ineffective” quadrant, characterized by low predicted bioactive benefits and moderate sugar levels. These findings challenge the reductionist “sugar-centric” view that all high-sugar fruits should be categorically avoided in T2DM management. Epidemiological and clinical evidence supports this nuanced perspective, suggesting that whole-fruit consumption—even of varieties with higher sugar content—can be associated with neutral or beneficial glycemic outcomes due to the protective influence of the fiber and bioactive matrix [62,63,64]. 

When extrapolating this scoring logic to fruits not covered in the present study—as requested by the current analytical framework—it is assumed that berries (e.g., *Vaccinium* and *Rubus* species) would represent the “Ideal Choice” (High Benefit/Low Risk) for diabetic consumers. These varieties are characterized by a high density of anthocyanins and flavanols—identified by our model as high-affinity ligands for metabolic targets—paired with a minimal glycemic load. However, this study intentionally focused on the five most prevalent staple fruits in the South Korean market rather than these “ideal” candidates. This choice was driven by the “clinical dilemma” these staple fruits present: their high commercial availability and significant sugar content require a far more nuanced, evidence-based evaluation than fruits that are naturally low in carbohydrates.

By providing a framework to address the metabolic trade-offs of these ubiquitous varieties, we empower consumers to manage the fruits they encounter most frequently in their daily diets. The development of such predictive, computation-driven frameworks to identify “Redemptive” or “Ideal” cultivars aligns with emerging global trends in precision nutrition [65] and the targeted computational screening of bioactives [66] for chronic disease management. This shift toward a variety-specific evaluation is essential for moving beyond traditional “one-size-fits-all” dietary guidelines [24].

### 4.4. Mechanistic Basis for Functional Classification

Hierarchical clustering of standardized phytochemical data (Figure 4) confirmed the chemical drivers underpinning the functional classification of the five Korean fruits. Grape was distinguished by its uniquely high resveratrol concentration, while dominant levels of hesperidin characterized orange. These key bioactive compounds are predicted to exert a dual antidiabetic mechanism: (i) inhibition of intestinal α-glucosidase, which slows the enzymatic breakdown of carbohydrates and attenuates postprandial glucose spikes, and (ii) activation of PPAR-γ, a nuclear receptor that enhances systemic insulin sensitivity and facilitates glucose uptake in peripheral tissues [67,68,69,70]. The co-occurrence of these effects illustrates that the metabolic impact of a fruit is a function not solely of sugar content but of the integrated actions of its phytochemical matrix.

Beyond the direct inhibition of α-glucosidase and the activation of PPAR-γ, the identified fruit cultivars may exert a broader influence on metabolic homeostasis through the incretin pathway. Recent studies emphasize that nutritional interventions can significantly improve metabolic health by modulating glucagon-like peptide-1 (GLP-1) concentrations, which enhances insulin sensitivity and alleviates metabolic dysregulation [71]. This mechanism is further supported by the emerging role of food-derived bioactives as inhibitors of Dipeptidyl peptidase-4 (DPP4)—the primary enzyme responsible for GLP-1 degradation [72]. The lead compounds identified in our study, such as quercetin and hesperidin, align with these findings as they have been increasingly recognized as natural multi-target modulators capable of protecting the incretin effect. This multifaceted ‘triple-pathway’ mechanism provides a robust mechanistic foundation for the ‘Redemptive Choices’ identified in our framework, suggesting that these fruits address both gut-level glucose absorption and systemic hormonal regulation.

### 4.5. Cultivar-Level Optimization

At the cultivar level, the ‘Campbell Early’ grape emerged as the optimal choice, achieving a Holistic Value Score (HVS = 9.5) that significantly outperformed ‘Shine Muscat’ (HVS = 3.9) and ‘Kyoho’ (HVS = 3.0) (Table 4, Figure 5). This outcome reflects a synergistic combination of elevated resveratrol density, moderate sugar levels, and a favorable micronutrient profile. The marked distinction between cultivars highlights a critical insight: even within a single fruit species, compositional variability can profoundly influence predicted antidiabetic potential [73,74].

The identification of ‘Campbell Early’ as the functionally superior choice—despite its status as the variety with the highest market price—highlights the most significant utility of our integrative framework. In the contemporary South Korean market, ‘Campbell Early’ is positioned as a premium variety; our analysis provides a mechanistic justification for this status by revealing that its phytochemical density justifies the economic investment. By treating market price as a “cost penalty” within the multi-criteria decision analysis, the framework effectively acts as a “Nutritional Return on Investment (ROI)” index. The fact that ‘Campbell Early’ achieved the highest HVS despite this economic penalty indicates that its predicted bioactivity is sufficiently potent to overcome the cost burden. This transforms the consumer experience from one based on subjective price-matching to one guided by a high-resolution decision-support tool. By quantitatively weighing both bioactive concentration and glycemic risk against real-world economic metrics, the framework identifies the cultivar that maximizes functional benefit while minimizing metabolic burden, offering an evidence-based blueprint for consumer guidance in diabetic nutrition.

## 5. Conclusions

This study establishes a novel, high-resolution integrative framework that shifts the evaluation of fruits for T2DM management from a carbohydrate-centric perspective to a multidimensional bioactivity assessment. By integrating in silico docking with measured phytochemical concentrations, we identified grapes and oranges (specifically cultivars like ‘Campbell Early’) as “Redemptive Choices” that provide a superior balance of antidiabetic bioactives against their inherent glycemic load.

However, these findings should be interpreted as an informed selection guide rather than a recommendation to increase total fruit consumption. Our results suggest that for T2DM patients, fruit selection should prioritize ‘quality over quantity’, selecting cultivars that maximize the ‘phytochemical-to-sugar’ ratio within prescribed dietary limits. While this framework provides a robust mechanistic blueprint for fruit evaluation, the experimental validations (in vitro and in vivo) outlined in our limitations remain a necessary prerequisite before these findings can be translated into formal clinical dietary guidelines. Ultimately, this research provides consumers with a decision-support tool that bridges the gap between molecular pharmacology and real-world nutritional choices.

In conclusion, this study provides an evidence-based in silico blueprint for fruit selection in T2DM management. By identifying ‘Redemptive Choices’ that maximize phytochemical ROI against glycemic risk, we empower consumers with a practical decision-support tool. While this model is verified against existing literature benchmarks, it serves as the critical pre-clinical foundation required to guide and derisk future in vitro and in vivo trials.

### Limitations

The predictive power of this study is rooted in its in silico and compositional nature, which, while a limitation, also constitutes its primary strength. Our model provides a robust, rational framework and generates clear, testable hypotheses, but does not constitute direct biological proof. To address this, the computational findings were cross-referenced with externally validated pharmacological benchmarks (Table 3), demonstrating that the predicted potency rankings exhibit high fidelity to established in vitro bioactivity.

The logical next step is to validate these findings in a biological context. We propose a two-stage pipeline beginning with in-vitro cell-based assays (e.g., in 3T3-L1 adipocytes) to directly measure the effect of top-ranked (‘Campbell Early’) versus lower-ranked (‘Shine Muscat’) grape extracts on glucose uptake and PPAR-γ target gene expression. Successful in-vitro results would then provide the rationale for a definitive preclinical in-vivo study in a diabetic mouse model (e.g., *db*/*db* mice), comparing the metabolic outcomes of diets supplemented with these extracts.

Furthermore, as a proof-of-concept study, this framework was focused on a strategic subset of five fruit types to ensure a high-resolution integration of bioactive potency and market metrics. While this limited scope allowed for a deep-dive analysis of staple fruits, we acknowledge that a broader database is required for comprehensive dietary guidance. This study serves as the critical and predictive blueprint to de-risk and guide future investigations, which will eventually expand this validated methodology to a wider array of global and seasonal produce.

## Figures and Tables

**Table 1:** General Nutritional and Mineral Composition of Fruit Samples per 100 g Weight (FW).

Variety		Glycemic Profile (g/100 g)				Proximate (g)			Vitamins				Minerals (mg)				
Fruit Sample	Country	Sugar (g)	Fiber (g)	GI	GL	Carbs (g)	Fats (g)	Protein (g)	Vit C (mg)	Vit K (µg)	*β*-car (µg)	Folate (ug)	K (mg)	Mg (mg)	Ca (mg)	Zn (mg)	Fe (mg)
Apple	Korea	11.14	2.70	36.00	2.45	13.58	0.77	0.20	1.41	0.00	9.00	3.00	107.00	3 ± 0.00	4.00	0.05	0.10
	India	9.53 ± 0.23	2.59 ± 0.15	38.00	6.00	9.53 ± 0.23	0.64 ± 0.04	0.29 ± 0.08	3.57 ± 0.58	3.65 ± 0.47	2.41 ± 2.13	3.04 ± 0.94	116 ± 12.9	8.09 ± 0.81	13.60 ± 3.20	0.09 ± 0.04	0.26 ± 0.02
Grapes	Korea	8.46	2.20	43.00	6.55	15.01	0.13	0.71	2.42	27.72	67.00	5.00	224.00	0.08	9.00	0.04	0.17
	India	10.02 ± 0.05	1.35 ± 0.14	53.00	11.00	10.02 ± 0.05	0.32 ± 0.02	0.76 ± 0.13	18.3 ± 2.32	3.65 ± 0.04	29.36 ± 8.37	8.69 ± 1.60	171 ± 26.50	6.7 ± 1.10	10.57 ± 2.03	0.05 ± 0.02	0.22 ± 0.04
Orange	Korea	10.17	1.50	40.00	4.70	12.80	0.11	0.99	52.94	0.00	38.00	19.00	154.0	9.00	11.00	0.04	0.11
	India	6.86 ± 0.006	1.29 ± 0.05	43.00	4.00	6.86 ± 0.06	0.13 ± 0.02	0.70 ± 0.12	42.72 ± 4.81	2.50 ± 0.75	31.94 ± 2.12	19.46 ± 1.09	164 ± 23.6	11.05 ± 0.52	19.52 ± 1.48	0.04 ± 0.01	0.81 ± 0.04
Peach	Korea	9.29	4.30	34.00	2.25	13.03	0.04	0.40	1.67	0.00	105.00	0.00	188.00	0.02	4.00	0.06	0.10
	India	6.95 ± 0.00	2.13 ± 0.00	42.00	5.00	6.95 ± 0.00	0.37 ± 0.00	0.86 ± 0.00	5.49 ± 0.00	4.4 ± 0.00	0.00	6.34 ± 0.00	281 ± 0.00	8.06 ± 0.00	6.98 ± 0.00	0.1 ± 0.00	0.35 ± 0.00
Pear	Korea	9.81	1.40	38.00	1.80	12.35	0.04	0.30	2.76	0.00	0.00	0.00	128.00	6.00	1.00	0.05	0.05
	India	7.39 ± 0.08	4.48 ± 0.08	38.00	4.00	7.39 ± 0.77	0.27 ± 0.04	0.36 ± 0.04	3.31 ± 0.9	12.57 ± 1.55	13.16 ± 1.78	5.28 ± 1.17	106 ± 6.60	7.61 ± 1.23	6.55 ± 1.39	0.07± 0.02	0.28 ± 0.01

**Note**: Fresh Values are presented as Mean (±Standard Deviation) where available. Where no standard deviation was reported in the source literature, only the mean value is shown. **Abbreviations:** GI, Glycemic Index; GL, Glycemic Load; β-car, Beta-carotene; Vit, Vitamin. **Minerals:** K, Potassium; Mg, Magnesium; Ca, Calcium; Zn, Zinc; Fe, Iron. **Units:** All glycemic and proximate values are expressed in g/100 g fresh weight unless otherwise specified.

**Table 2:** Binding Energies (kcal/mol) of key bioactive compounds against the active sites of NOX2, α-glucosidase, and PPAR-γ as predicted by AutoDock Vina.

Bioactive Compound	Chemical Class	NOX2 (PDB: 3A1F)	α-Glucosidase (PDB: 7KAD)	PPAR-γ (PDB: 2PRG)
Chlorogenic Acid	Phenolic acids	−6	−7.9	−7.5
Resveratrol	Stillbenoid	−5	−8.2	−6.9
Hesperidin	Flavanone	** −6.7 **	** −9.7 **	** −9.3 **
Catechins	Flavanol	−5.6	−8.4	−7.4
Epicatechins	Flavanol	−5.7	−7.6	−7.4
Quercetin	Flavonol	−5.8	−8.3	−7.8

Values represent the binding affinity (in kcal/mol) for the top-ranked docking pose (the pose with the most negative binding energy) as calculated by AutoDock Vina. A more negative value indicates a stronger predicted binding interaction.

**Table 4:** Comparative analysis of key performance indicators for three Korean grape cultivars.

Cultivar	Sugar (g/100 g)	Resveratrol (mg/100 g)	Average $ Price (won/100 g)	Micronutrient Profile Score	Holistic Score Value
Campbell Early	8.46	63.46 ± 14.11	2455.33	3	**9.5**
Shine Muscat	15.29	46.72 ± 13.21	761.67	1	3.9
Kyoho	12.32	37.86 ± 4.35	2433	2	3

## Data Availability

The original contributions presented in the study are included in the article/Supplementary Materials; further inquiries will be made available on request.

## Supplementary Materials

The following supporting information can be downloaded at: https://www.mdpi.com/article/10.3390/foods15050797/s1. Table S1: Comprehensive Nutritional and Mineral Composition of All 10 Fruit Samples, Table S2: Comprehensive Phytochemical Composition of All 10 Fruit Samples, Table S3: Detailed Molecular Docking Results, Figure S1: Scree Plot for the Principal Component Analysis. Figure S2: Full Hierarchical Clustering Heatmap of All 10 Fruit Samples, Figure S3: (**A**) Docking visualization for 3A1F (**B**) Docking visualization for 7KAD.

## References

[1] Pan C., Cao B., Fang H., Liu Y., Zhang S., Luo W., Wu Y. (2025). Global burden of diabetes mellitus 1990–2021: Epidemiological trends, geospatial disparities, and risk factor dynamics. Front. Endocrinol..

[2] Zimmet P.Z., Magliano D.J., Herman W.H., Shaw J.E. (2014). Diabetes: A 21st century challenge. Lancet Diabetes Endocrinol..

[3] International Diabetes Federation (2025). IDF Diabetes Atlas.

[4] Ruze R., Liu T., Zou X., Song J., Chen Y., Xu R., Yin X., Xu Q. (2023). Obesity and type 2 diabetes mellitus: Connections in epidemiology, pathogenesis, and treatments. Front. Endocrinol..

[5] Westman E.C. (2021). Type 2 diabetes mellitus: A pathophysiologic perspective. Front. Nutr..

[6] Dludla P.V., Mabhida S.E., Ziqubu K., Nkambule B.B., Mazibuko-Mbeje S.E., Hanser S., Basson A.K., Pheiffer C., Kengne A.P. (2023). Pancreatic β-cell dysfunction in type 2 diabetes: Implications of inflammation and oxidative stress. World J. Diabetes.

[7] Wu Q., Gao Z.-J., Yu X., Wang P. (2022). Dietary regulation in health and disease. Signal Transduct. Target. Ther..

[8] (2023). Evidence-based European recommendations for the dietary management of diabetes. Diabetologia.

[9] Feinman R.D., Pogozelski W.K., Astrup A., Bernstein R.K., Fine E.J., Westman E.C., Accurso A., Frassetto L., Gower B.A., McFarlane S.I. (2015). Dietary carbohydrate restriction as the first approach in diabetes management: Critical review and evidence base. Nutrition.

[10] Singh M.K., Han S., Ju S., Ranbhise J.S., Akter S., Kim S.S., Kang I. (2025). Fruit Carbohydrates and Their Impact on the Glycemic Index: A Study of Key Determinants. Foods.

[11] Clemente-Suárez V.J., Mielgo-Ayuso J., Martín-Rodríguez A., Ramos-Campo D.J., Redondo-Flórez L., Tornero-Aguilera J.F. (2022). The burden of carbohydrates in health and disease. Nutrients.

[12] Sánchez M., Laca A., Laca A., Díaz M. (2021). Value-added products from fruit and vegetable wastes: A review. CLEAN–Soil Air Water.

[13] Ley S.H., Hamdy O., Mohan V., Hu F.B. (2014). Prevention and management of type 2 diabetes: Dietary components and nutritional strategies. Lancet.

[14] Hei Karen Lau K. (2022). Nutrition therapy for adults with diabetes or prediabetes. ADCES Pract..

[15] Nicholls J. (2022). Perspective: The Glycemic Index Falls Short as a Carbohydrate Food Quality Indicator to Improve Diet Quality. Front. Nutr..

[16] Shrinet K., Singh R.K., Chaurasia A.K., Tripathi A., Kumar A. (2021). Bioactive Compounds and Their Future Therapeutic Applications. Natural Bioactive Compounds.

[17] Naz R., Saqib F., Awadallah S., Wahid M., Latif M.F., Iqbal I., Mubarak M.S. (2023). Food Polyphenols and Type II Diabetes Mellitus: Pharmacology and Mechanisms. Molecules.

[18] Williamson G. (2013). Possible effects of dietary polyphenols on sugar absorption and digestion. Mol. Nutr. Food Res..

[19] Pandey K.B., Rizvi S.I. (2009). Plant polyphenols as dietary antioxidants in human health and disease. Oxidative Med. Cell. Longev..

[20] Egbuna C., Awuchi C.G., Kushwaha G., Rudrapal M., Patrick-Iwuanyanwu K.C., Singh O., Odoh U.E., Khan J., Jeevanandam J., Kumarasamy S. (2021). Bioactive compounds effective against type 2 diabetes mellitus: A systematic review. Curr. Top. Med. Chem..

[21] Arabshomali A., Bazzazzadehgan S., Mahdi F., Shariat-Madar Z. (2023). Potential benefits of antioxidant phytochemicals in type 2 diabetes. Molecules.

[22] Xiao J., Kai G., Yamamoto K., Chen X. (2013). Advance in dietary polyphenols as α-glucosidases inhibitors: A review on structure-activity relationship aspect. Crit. Rev. Food Sci. Nutr..

[23] AL-Ishaq R.K., Abotaleb M., Kubatka P., Kajo K., Büsselberg D. (2019). Flavonoids and Their Anti-Diabetic Effects: Cellular Mechanisms and Effects to Improve Blood Sugar Levels. Biomolecules.

[24] Kussmann M., Abe Cunha D.H., Berciano S. (2023). Bioactive compounds for human and planetary health. Front. Nutr..

[25] Shashirekha M., Mallikarjuna S., Rajarathnam S. (2015). Status of bioactive compounds in foods, with focus on fruits and vegetables. Crit. Rev. Food Sci. Nutr..

[26] Pandohee J., Kyereh E., Kulshrestha S., Xu B., Mahomoodally M.F. (2023). Review of the recent developments in metabolomics-based phytochemical research. Crit. Rev. Food Sci. Nutr..

[27] Damián-Medina K., Salinas-Moreno Y., Milenkovic D., Figueroa-Yáñez L., Marino-Marmolejo E., Higuera-Ciapara I., Vallejo-Cardona A., Lugo-Cervantes E. (2020). In silico analysis of antidiabetic potential of phenolic compounds from blue corn (*Zea mays* L.) and black bean (*Phaseolus vulgaris* L.). Heliyon.

[28] Ali A., Cottrell J.J., Dunshea F.R. (2023). Antioxidant, alpha-glucosidase inhibition activities, in silico molecular docking and pharmacokinetics study of phenolic compounds from native australian fruits and spices. Antioxidants.

[29] Olaokun O.O., Manonga S.A., Zubair M.S., Maulana S., Mkolo N.M. (2022). Molecular Docking and Molecular Dynamics Studies of Antidiabetic Phenolic Compound Isolated from Leaf Extract of *Englerophytum magalismontanum* (Sond.) TD Penn. Molecules.

[30] Dallakyan S., Olson A.J. (2014). Small-Molecule Library Screening by Docking with PyRx. Chemical Biology: Methods and Protocols.

[31] Agu P.C., Afiukwa C.A., Orji O.U., Ezeh E.M., Ofoke I.H., Ogbu C.O., Ugwuja E.I., Aja P.M. (2023). Molecular docking as a tool for the discovery of molecular targets of nutraceuticals in diseases management. Sci. Rep..

[32] Nagre K., Singh N., Ghoshal C., Tandon G., Iquebal M.A., Nain T., Bana R.S., Meena A. (2023). Probing the potential of bioactive compounds of millets as an inhibitor for lifestyle diseases: Molecular docking and simulation-based approach. Front. Nutr..

[33] (2025). Korean Food Composition Database.

[34] Longvah T., Anantan I., Bhaskarachary K., Venkaiah K., Longvah T. (2017). Indian Food Compositions Table.

[35] Mirzaei A., Mirzaei A., Najjar Khalilabad S., Askari V.R., Baradaran Rahimi V. (2023). Promising influences of hesperidin and hesperetin against diabetes and its complications: A systematic review of molecular, cellular, and metabolic effects. Excli J..

[36] Peng J., Lu C., Luo Y., Su X., Li S., Ho C.-T. (2024). Hypoglycemic effects and associated mechanisms of resveratrol and related stilbenes in diet. Food Funct..

[37] Aryal D., Joshi S., Thapa N.K., Chaudhary P., Basaula S., Joshi U., Bhandari D., Rogers H.M., Bhattarai S., Sharma K.R. (2024). Dietary phenolic compounds as promising therapeutic agents for diabetes and its complications: A comprehensive review. Food Sci. Nutr..

[38] Tao X., Huang Y., Wang C., Chen F., Yang L., Ling L., Che Z., Chen X. (2020). Recent developments in molecular docking technology applied in food science: A review. Int. J. Food Sci. Technol..

[39] Asiamah I., Obiri S.A., Tamekloe W., Armah F.A., Borquaye L.S. (2023). Applications of molecular docking in natural products-based drug discovery. Sci. Afr..

[40] Shanak S., Naim S.A., AlArdah B., Bassalat N., Zaid H. (2025). Ligand-protein docking of phytochemicals in their plausible binding to alpha-amylase and alpha-glucosidase enzymes and ligand bioavailability. Food Chem. Adv..

[41] (2025). BIOVIA Discovery Studio Visualizer, v25.1.0.24284.

[42] Proença C., Freitas M., Ribeiro D., Oliveira E.F., Sousa J.L., Tomé S.M., Ramos M.J., Silva A.M., Fernandes P.A., Fernandes E. (2017). α-Glucosidase inhibition by flavonoids: An in vitro and in silico structure–activity relationship study. J. Enzym. Inhib. Med. Chem..

[43] Kaliaperumal K., Zhang L., Gao L., Xiong Q., Liang Y., Jiang Y., Zhang J. (2023). Insight into the inhibitory mechanisms of hesperidin on α-glucosidase through kinetics, fluorescence quenching, and molecular docking studies. Foods.

[44] Ahmadian M., Suh J.M., Hah N., Liddle C., Atkins A.R., Downes M., Evans R.M. (2013). PPARγ signaling and metabolism: The good, the bad and the future. Nat. Med..

[45] Brown O.I., Bridge K.I., Kearney M.T. (2021). Nicotinamide adenine dinucleotide phosphate oxidases in glucose homeostasis and diabetes-related endothelial cell dysfunction. Cells.

[46] Fujimura Y., Kawano C., Maeda-Murayama A., Nakamura A., Koike-Miki A., Yukihira D., Hayakawa E., Ishii T., Tachibana H., Wariishi H. (2017). A chemometrics-driven strategy for the bioactivity evaluation of complex multicomponent systems and the effective selection of bioactivity-predictive chemical combinations. Sci. Rep..

[47] National Library of Medicine. PUBCHEM. USA. National Center for Biotechnology Information. PubChem Compound Summary Database; National Library of Medicine: Bethesda, MD, USA. 20894. https://pubchem.ncbi.nlm.nih.gov.

[48] Dallakyan S. (2010). PyRx-Python Prescription, version 0.8.

[49] Agarwal R., Smith J.C. (2023). Speed vs accuracy: Effect on ligand pose accuracy of varying box size and exhaustiveness in AutoDock vina. Mol. Inform..

[50] Yusuf D., Davis A.M., Kleywegt G.J., Schmitt S. (2008). An alternative method for the evaluation of docking performance: RSR vs RMSD. J. Chem. Inf. Model..

[51] Patil R., Das S., Stanley A., Yadav L., Sudhakar A., Varma A. (2010). Optimized Hydrophobic Interactions and Hydrogen Bonding at the Target-Ligand Interface Leads the Pathways of Drug-Designing. PLoS ONE.

[52] Talevi A. (2015). Multi-target pharmacology: Possibilities and limitations of the “skeleton key approach” from a medicinal chemist perspective. Front. Pharmacol..

[53] Pang Z., Lu Y., Zhou G., Hui F., Xu L., Viau C., Spigelman A.F., MacDonald P.E., Wishart D.S., Li S. (2024). MetaboAnalyst 6.0: Towards a unified platform for metabolomics data processing, analysis and interpretation. Nucleic Acids Res..

[54] Metsalu T., Vilo J. (2015). ClustVis: A web tool for visualizing clustering of multivariate data using Principal Component Analysis and heatmap. Nucleic Acids Res..

[55] Steffen Y., Gruber C., Schewe T., Sies H. (2008). Mono-O-methylated flavanols and other flavonoids as inhibitors of endothelial NADPH oxidase. Arch. Biochem. Biophys..

[56] Sakata K., Hirose Y., Qiao Z., Tanaka T., Mori H. (2003). Inhibition of inducible isoforms of cyclooxygenase and nitric oxide synthase by flavonoid hesperidin in mouse macrophage cell line. Cancer Lett..

[57] Zaidi S.Y.R., Ahmed A., Sanghvi G., Roopashree R., Kashyap A., Mohammed N.K., Niaz B., Saeed F., Jamil F., Akhter M.N. (2025). Exploring the In Vitro Anti-Inflammatory Effect of Citrus Fruit Hesperidin Supplementation. Food Sci. Nutr..

[58] Calleri E., Pochetti G., Dossou K.S., Laghezza A., Montanari R., Capelli D., Prada E., Loiodice F., Massolini G., Bernier M. (2014). Resveratrol and its metabolites bind to PPARs. ChemBioChem.

[59] Khattak K.F., Rahman T.R. (2015). Effect of geographical distributions on the nutrient composition, phytochemical profile and antioxidant activity of Morus nigra. Pak. J. Pharm. Sci..

[60] Zhang Q., Li M., Zhou B., Zhang J., Wei Q. (2023). Quantitative Analysis of Relationship Between Fruit Quality of ‘Fuji’ Apple and Environmental Factors: A Case Study of the Loess Plateau Production Region. Erwerbs-Obstbau.

[61] Zhong H., Xu J., Yang M., Hussain M., Liu X., Feng F., Guan R. (2023). Protective effect of anthocyanins against neurodegenerative diseases through the microbial-intestinal-brain axis: A critical review. Nutrients.

[62] Su J., Qin Y., Pan X.Q., Shen C., Gao Y., Pan E.C., Zhang Y.Q., Zhou J.Y., Wu M. (2019). Association between fresh fruit consumption and glycemic control in patients with type 2 diabetes. Zhonghua Liu Xing Bing Xue Za Zhi.

[63] Muraki I., Imamura F., Manson J.E., Hu F.B., Willett W.C., van Dam R.M., Sun Q. (2013). Fruit consumption and risk of type 2 diabetes: Results from three prospective longitudinal cohort studies. Bmj.

[64] Christensen A.S., Viggers L., Hasselström K., Gregersen S. (2013). Effect of fruit restriction on glycemic control in patients with type 2 diabetes–a randomized trial. Nutr. J..

[65] Berry S.E., Valdes A.M., Drew D.A., Asnicar F., Mazidi M., Wolf J., Capdevila J., Hadjigeorgiou G., Davies R., Al Khatib H. (2020). Human postprandial responses to food and potential for precision nutrition. Nat. Med..

[66] Barabási A.-L., Menichetti G., Loscalzo J. (2020). The unmapped chemical complexity of our diet. Nat. Food.

[67] Zhang A.J., Rimando A.M., Mizuno C.S., Mathews S.T. (2017). α-Glucosidase inhibitory effect of resveratrol and piceatannol. J. Nutr. Biochem..

[68] Rasouli H., Hosseini-Ghazvini S.M., Adibi H., Khodarahmi R. (2017). Differential α-amylase/α-glucosidase inhibitory activities of plant-derived phenolic compounds: A virtual screening perspective for the treatment of obesity and diabetes. Food Funct..

[69] Bahadoran Z., Mirmiran P., Azizi F. (2013). Dietary polyphenols as potential nutraceuticals in management of diabetes: A review. J. Diabetes Metab. Disord..

[70] Rabbani N., Xue M., Weickert M.O., Thornalley P.J. (2021). Reversal of Insulin Resistance in Overweight and Obese Subjects by trans-Resveratrol and Hesperetin Combination-Link to Dysglycemia, Blood Pressure, Dyslipidemia, and Low-Grade Inflammation. Nutrients.

[71] Yang M., Fu X., Liu S., Pan Y., Cai H., Wang J., Feng F., Zhao M. (2025). Synthesized capric-lauric acid structural lipids improve metabolic health in obesity: Roles of antimicrobial activity, systemic inflammation and gut microbiota remodeling. Food Biosci..

[72] Shen F., Deng Q., Song Y., He G., Chu X., Zhao M., Du J., Feng F., Zhang X., Zhong H. (2025). Amelioration of Wheat Peptides on Diet-Induced Hyperglycemia via Multi-Target Manner: Integrated Modulation of Inflammation, Lipid Metabolism, Gut Microbiota, and DPP-IV Inhibition. Food Funct..

[73] Ferla G., Mura B., Falasco S., Caputo P., Matarazzo A. (2024). Multi-Criteria Decision Analysis (MCDA) for sustainability assessment in food sector. A systematic literature review on methods, indicators and tools. Sci. Total Environ..

[74] Ali B.M., Andersson M.G., van den Borne B.H.P., Focker M., van der Fels-Klerx H.J. (2022). Multi-Criteria Decision Analysis in Food Safety Risk Management: The Case of Dioxins in Baltic Fish. Foods.

