# Supplementary material for: Integrating Phytochemical Bioactivity and Glycemic Risk to Evaluate Fruits for Type 2 Diabetes Management: A Korean Market Perspective
Source: Foods. 2026 Feb 24;15(5):797. doi: 10.3390/foods15050797 (PMC12984167; doi:10.3390/foods15050797)
Supplement: Supplementary file 1 [file foods-15-00797-s001.zip › foods-4136867-Supplementary Materials.pdf]

List of Supplementary Data and Figures

Supplementary Table S1: Comprehensive Nutritional and Mineral Composition of All 10 Fruit Samples. (originally a large table S1, sectioned into a,b,c for readability)

Supplementary Table S1a. Comprehensive Nutritional and Glycemic Composition of Initial Fruit Screening Set (n=10)

Fruit Variety	Origin	Total Sugar (g/100g)	Total Carbs g/100g)	Fiber (g/100g)	Protein (g/100g)	Fat (g/100g)	Glycemic Index (GI)	Glycemic Load (GL)
**Apple**	Korea	11.14 ± 0.00	13.58 ± 0.00	2.70 ± 0.00	0.20 ± 0.00	0.77 ± 0.00	36 ± 0	2.45 ± 0.00
	India	9.53 ± 0.23	9.53 ± 0.23	2.59 ± 0.15	0.29 ± 0.08	0.64 ± 0.04	38 ± 0	6.00 ± 0.00
**Grapes**	Korea	8.46 ± 0.00	15.01 ± 0.00	2.20 ± 0.00	0.71 ± 0.00	0.13 ± 0.00	43 ± 0	6.55 ± 0.00
	India	10.02 ± 0.05	10.02 ± 0.05	1.35 ± 0.14	0.76 ± 0.13	0.32 ± 0.02	53 ± 0	11.00 ± 0.00
**Orange**	Korea	10.17 ± 0.00	12.80 ± 0.00	1.50 ± 0.00	0.99 ± 0.00	0.11 ± 0.00	40 ± 0	4.70 ± 0.00
	India	6.86 ± 0.06	6.86 ± 0.06	1.29 ± 0.05	0.70 ± 0.12	0.13 ± 0.02	43 ± 0	4.00 ± 0.00
**Peach**	Korea	9.29 ± 0.00	13.03 ± 0.00	4.30 ± 0.00	0.40 ± 0.00	0.04 ± 0.00	34 ± 0	2.25 ± 0.00
	India	6.95 ± 0.00	6.95 ± 0.00	2.13 ± 0.00	0.86 ± 0.00	0.37 ± 0.00	42 ± 0	5.00 ± 0.00
**Pear**	Korea	9.81 ± 0.00	12.35 ± 0.00	1.40 ± 0.00	0.30 ± 0.00	0.04 ± 0.00	38 ± 0	1.80 ± 0.00
	India	7.39 ± 0.08	7.39 ± 0.77	4.48 ± 0.08	0.36 ± 0.04	0.27 ± 0.04	38 ± 0	4.00 ± 0.00

Supplementary Table S1b. Detailed Sugar Profiles of Fruit Varieties from Korean and Indian Markets

Fruit Variety	Origin	Free Glucose (g/100g)	Total Glucose (g/100g)	Free Fructose (g/100g)	Total Fructose (g/100g)	Sucrose (g/100g)
**Apple**	Korea	2.62 ± 0.00	3.75 ± 0.00	6.37 ± 0.00	7.50 ± 0.00	2.15 ± 0.00
	India	1.03 ± 0.02	1.10 ± 0.03	8.36 ± 0.23	8.43 ± 0.24	0.14 ± 0.02
**Grapes**	Korea	3.76 ± 0.00	3.76 ± 0.00	4.70 ± 0.00	4.70 ± 0.00	0.00 ± 0.00
	India	2.77 ± 0.03	2.85 ± 0.05	7.10 ± 0.02	7.18 ± 0.04	0.15 ± 0.03
**Orange**	Korea	2.79 ± 0.00	4.99 ± 0.00	3.21 ± 0.00	5.41 ± 0.00	4.18 ± 0.00

	India	1.21 ± 0.02	2.68 ± 0.04	2.86 ± 0.03	4.33 ± 0.05	2.79 ± 0.03
**Peach**	Korea	4.20 ± 0.00	4.77 ± 0.00	4.00 ± 0.00	4.57 ± 0.00	1.09 ± 0.00
	India	0.89 ± 0.00	3.32 ± 0.00	1.15 ± 0.00	3.58 ± 0.00	4.61 ± 0.00
**Pear**	Korea	4.44 ± 0.00	4.71 ± 0.00	4.86 ± 0.00	5.13 ± 0.00	0.51 ± 0.00
	India	2.74 ± 0.30	3.33 ± 0.31	3.51 ± 0.21	4.10 ± 0.22	1.12 ± 0.01

Supplementary Table S1c. Vitamin and Mineral Composition of Korean and Indian Fruit Cultivars

Fruit	Origin	Vit C (mg)	β -car (µg)	Folate (µg)	Vit K (µg)	K (mg)	Mg (mg)	Ca (mg)	Fe (mg)	Zn (mg)
**Apple**	Korea	1.41 ± 0	9.0 ± 0	3.0 ± 0	3.65 ± 0	107 ± 0	3.0 ± 0	4.0 ± 0	0.1 ± 0	0.05 ± 0
	India	3.57 ± 0.6	2.4 ± 2.1	3.0 ± 0.9	0.0 ± 0	116 ± 12	8.1 ± 0.8	13.7 ± 3	0.2 ± 0.02	0.09 ± 0.04
**Grapes**	Korea	2.42 ± 0	67 ± 0	5.0 ± 0	27.7 ± 0	224 ± 0	0.1 ± 0	9.0 ± 0	0.17 ± 0	0.04 ± 0
	India	18.3 ± 2.3	29.4 ± 8.4	8.7 ± 1.6	0.0 ± 0	171 ± 26	6.9 ± 1.1	10.6 ± 2	0.2 ± 0.04	0.05 ± 0.02
**Orange**	Korea	52.9 ± 0	38 ± 0	19 ± 0	0.0 ± 0	154 ± 0	9.0 ± 0	11 ± 0	0.11 ± 0	0.04 ± 0
	India	42.7 ± 4.8	31.9 ± 2.1	19.5 ± 1.1	0.0 ± 0	164 ± 23	11 ± 0.5	19.5 ± 1.5	0.8 ± 0.04	0.04 ± 0.01
**Peach**	Korea	1.67 ± 0	105 ± 0	0.0 ± 0	0.0 ± 0	188 ± 0	0.02 ± 0	4.0 ± 0	0.1 ± 0	0.06 ± 0
	India	5.49 ± 0	0.0 ± 0	6.3 ± 0	0.0 ± 0	281 ± 0	8.1 ± 0	7.0 ± 0	0.35 ± 0	0.1 ± 0
**Pear**	Korea	2.76 ± 0	0.0 ± 0	0.0 ± 0	0.0 ± 0	128 ± 0	6.0 ± 0	1.0 ± 0	0.05 ± 0	0.05 ± 0
	India	3.31 ± 0.9	13.2 ± 1.8	5.3 ± 1.2	0.0 ± 0	106 ± 6.6	7.6 ± 1.2	6.6 ± 1.4	0.28 ± 0.1	0.07 ± 0.02

Calculations used -

Total Glucose = [Free Glucose] + ([Sucrose] × 0.527)

Total Fructose = [Free Fructose] + ([Sucrose] × 0.527)

Total glucose and total fructose concentrations were calculated to account for the sugars present in sucrose. The amount of glucose and fructose liberated from the hydrolysis of sucrose was calculated by multiplying the sucrose concentration by a conversion factor of 0.527. This factor was derived from the ratio of the molar masses of glucose (180.16 g/mol) and sucrose (342.30 g/mol), with values obtained from the PubChem database (PubChem CIDs: 5793 and 5988, respectively)

Supplementary Table S2: Comprehensive Phytochemical Composition of All 10 Fruit Samples.

Fruit Sample	Country of origin	Total Phenolic Content (mg GAE/100g)	Chlorogenic Acid (mg/100g)	Resveratrol (mg/100g)	Hesperidin (mg/100g)	Catechins (mg/100g)	Epicatechins (mg/100g)	Quercetin(mg/100g)
Apple	Korea	113.00	17.68	0.00	0.00	0.00	2.33	0.00
	India	35.81± 4.13	1.63 ± 0.13	0.00	0.00	1.21 ± 0.17	7.99 ± 0.64	0.87 ± 0.07
Grapes	Korea	129.57 ± 1.01	0.00	63.46 ± 14.11	0.00	4.17	0.48	9.63
	India	144 ± 10.00	0.63 ± 0.08	0.04 ± 0.01	0.02 ± 0.01	11.04 ± 1.47	7.83 ± 0.45	0.70 ± 0.06
Orange	Korea	102.4 ± 8.6	0.51	0.00	46.52	0.00	0.00	0.36
	India	54.73 ± 9.78	0.42 ± 0.02	0.00	24.13 ± 2.65	0.00	0.00	1.7 ± .004
Peach	Korea	15.86 ± 0.29	13.30	0.00	0.00	0.00	0.00	1.15
	India	279.00	0.00	0.00	0.00	2.33	5.46	0.00
Pear	Korea	38.7 ± 2.30	3.92	0.00	0.00	0.00	0.00	0.00
	India	1050 ± 1.95	1.27 ± 0.02	0.00	0.00	0.04 ± 0.01	1.09 ± 0.36	1.56 ± 0.25

Values are presented as Mean (± Standard Deviation) where available. Where no standard deviation was reported in the source literature, only the mean value is shown.

Supplementary Table S3: Detailed Molecular Docking Results

S3a. Comprehensive Binding Energy Profiles and RMSD Values for PPAR-γ (PDB: 2PRG)

Bioactive Compound (CID)	Pose #	Binding Affinity (ΔG, kcal/mol)	RMSD (L.B., Å)	RMSD (U.B., Å)
**Hesperidin (10621)**	**1**	**−9.3**	**0.000**	**0.000**
	2	−9.3	2.191	3.496
	3	−9.3	3.947	7.965
	4	−9.3	1.936	3.076
	5	−9.0	1.532	2.320
	6	−8.8	3.029	6.853
	7	−8.7	3.020	8.168
	8	−8.6	9.697	14.441
	9	−8.6	3.219	8.835
**Quercetin (5280343)**	**1**	**−7.8**	**0.000**	**0.000**
	2	−7.2	1.578	7.018
	3	−7.1	15.890	17.279
	4	−7.0	1.721	3.288
	5	−6.9	16.372	19.312
	6	−6.8	18.080	18.865
	7	−6.8	15.374	18.123
	8	−6.8	17.228	18.364
	9	−6.8	1.721	3.288
**Chlorogenic Acid (1794427)**	**1**	**−7.5**	**0.000**	**0.000**
	2	−7.4	1.865	2.467
	3	−7.4	2.677	7.922
	4	−7.2	4.235	9.877
	5	−7.2	16.235	17.514
	6	−7.2	2.209	7.887
	7	−7.1	15.026	16.911
	8	−7.0	12.965	14.641
	9	−7.0	2.190	3.893
**Resveratrol (445154)**	**1**	**−6.9**	**0.000**	**0.000**
	2	−6.9	0.084	2.014
	3	−6.8	1.245	7.596
	4	−6.7	1.266	7.467
	5	−6.7	9.292	11.260
	6	−6.6	9.614	11.733
	7	−6.5	13.937	16.940
	8	−6.3	12.535	13.753

	9	−6.0	13.529	15.949
**(−)-Epicatechin (72276)**	**1**	**−7.4**	**0.000**	**0.000**
	2	−6.9	8.913	12.152
	3	−6.9	2.337	4.275
	4	−6.8	16.737	18.298
	5	−6.8	16.650	17.944
	6	−6.7	1.913	7.020
	7	−6.7	16.770	19.418
	8	−6.6	7.027	9.574
	9	−6.6	2.863	3.484
**(+)-Catechin (9064)**	**1**	**−7.4**	**0.000**	**0.000**
	2	−7.1	2.565	2.663
	3	−7.0	1.396	6.859
	4	−6.9	16.080	19.013
	5	−6.9	15.255	18.125
	6	−6.8	17.678	18.710
	7	−6.5	16.447	18.931
	8	−6.5	7.788	10.931
	9	−6.5	16.518	18.827

S3b. Comprehensive Binding Energy Profiles and RMSD Values for NOX2 (PDB: 3A1F)

**Bioactive Compound (CID)**	**Pose #**	**Binding Affinity (ΔG, kcal/mol)**	**RMSD (L.B., Å)**	**RMSD (U.B., Å)**
**Hesperidin (10621)**	**1**	**−6.7**	**0.000**	**0.000**
	2	−6.6	2.448	5.039
	3	−6.6	1.714	2.721
	4	−6.4	1.666	4.339
	5	−6.3	2.227	4.766
	6	−6.3	1.286	1.772
	7	−6.2	1.990	3.531
	8	−5.8	1.566	2.413
	9	−5.6	2.066	8.036
**Chlorogenic Acid (1794427)**	**1**	**−6.0**	**0.000**	**0.000**
	2	−6.0	2.001	3.397
	3	−6.0	3.411	6.265
	4	−5.9	3.561	7.945
	5	−5.9	1.683	2.146
	6	−5.6	4.868	7.307
	7	−5.6	3.534	7.378
	8	−5.6	4.205	5.847

	9	−5.4	2.396	7.048
**Quercetin (5280343)**	**1**	**−5.8**	**0.000**	**0.000**
	2	−5.7	4.414	7.094
	3	−5.5	2.604	6.846
	4	−5.5	1.618	6.550
	5	−5.5	24.145	26.385
	6	−5.4	2.646	6.641
	7	−5.3	3.700	4.525
	8	−5.1	3.167	6.865
	9	−5.1	2.190	2.986
**(−)-Epicatechin (72276)**	**1**	**−5.7**	**0.000**	**0.000**
	2	−5.5	24.578	26.255
	3	−5.4	1.479	1.854
	4	−5.4	25.046	26.790
	5	−5.4	24.797	26.549
	6	−5.3	24.039	25.655
	7	−5.2	2.336	7.252
	8	−5.1	2.554	7.392
	9	−5.1	24.109	25.818
**(+)-Catechin (9064)**	**1**	**−5.6**	**0.000**	**0.000**
	2	−5.4	2.244	6.305
	3	−5.3	2.181	7.677
	4	−5.2	2.546	7.417
	5	−5.2	2.188	3.544
	6	−5.2	24.183	25.855
	7	−5.1	24.309	26.196
	8	−4.9	24.199	25.623
	9	−4.9	3.799	5.377
**Resveratrol (445154)**	**1**	**−5.0**	**0.000**	**0.000**
	2	−4.9	5.724	8.710
	3	−4.9	5.712	8.945
	4	−4.9	8.505	10.909
	5	−4.8	8.545	10.923
	6	−4.6	5.450	10.386
	7	−4.6	5.539	10.438
	8	−4.6	5.978	8.717
	9	−4.5	6.946	8.669

S3c. Comprehensive Binding Energy Profiles and RMSD Values for α-Glucosidase (PDB: 7KAD)

Bioactive Compound (CID)	Pose #	Binding Affinity (ΔG, kcal/mol)	RMSD (L.B., Å)	RMSD (U.B., Å)
**Hesperidin (10621)**	**1**	**−9.7**	**0.000**	**0.000**
	2	−9.6	5.303	7.575
	3	−9.4	1.652	2.296
	4	−9.3	5.541	11.285
	5	−9.1	2.370	9.317
	6	−9.1	1.890	9.409
	7	−8.9	5.956	11.131
	8	−8.9	8.945	14.130
	9	−8.8	1.576	9.797
**(+)-Catechin (9064)**	**1**	**−8.4**	**0.000**	**0.000**
	2	−7.2	1.490	2.301
	3	−7.1	2.917	5.404
	4	−7.0	3.457	5.362
	5	−7.0	1.685	6.761
	6	−7.0	3.342	7.517
	7	−7.0	3.566	9.320
	8	−6.9	3.465	5.646
	9	−6.9	3.104	4.954
**Quercetin (5280343)**	**1**	**−8.3**	**0.000**	**0.000**
	2	−8.1	0.054	2.332
	3	−8.0	1.524	3.257
	4	−7.9	4.299	6.443
	5	−7.8	3.555	5.265
	6	−7.8	4.071	8.808
	7	−7.8	5.374	9.372
	8	−7.5	3.803	6.173
	9	−7.4	1.721	6.896
**Resveratrol (445154)**	**1**	**−8.2**	**0.000**	**0.000**
	2	−8.1	0.054	2.332
	3	−7.9	1.404	6.697
	4	−7.9	1.482	6.742
	5	−7.8	3.555	5.265
	6	−7.6	4.299	6.443
	7	−7.6	1.524	3.257
	8	−7.5	1.128	7.552
	9	−7.4	1.721	6.896
**Chlorogenic Acid (1794427)**	**1**	**−7.9**	**0.000**	**0.000**
	2	−7.6	3.726	5.049
	3	−7.5	1.128	7.552
	4	−7.4	1.125	7.316

	5	−7.3	1.380	2.580
	6	−7.3	3.495	5.294
	7	−7.3	3.490	4.974
	8	−7.2	3.379	5.658
	9	−7.2	1.382	6.772
**(−)-Epicatechin (72276)**	**1**	**−7.6**	**0.000**	**0.000**
	2	−7.4	1.125	7.316
	3	−7.3	1.380	2.580
	4	−7.3	3.474	7.245
	5	−7.2	5.021	6.985
	6	−7.2	4.162	8.498
	7	−7.2	7.281	10.984
	8	−7.2	18.610	20.766
	9	−7.1	2.856	7.104

Notes: Pose 1 represents the conformation with the highest predicted binding affinity (ΔG). **RMSD (Root Mean Square Deviation)** values are provided as Lower Bound (L.B.) and Upper Bound (U.B.), calculated relative to the top-ranked pose (Pose 1). **ΔG:** Gibbs free energy, expressed in kcal/mol. **CID:** PubChem Compound Identification number. All docking simulations were executed using AutoDock Vina within the PyRx interface with an exhaustiveness setting of 8. Bolded values indicate the lead pose utilized for mechanistic and structural analysis in the main manuscript.

Supplementary Figure S1: Scree Plot for the Principal Component Analysis.

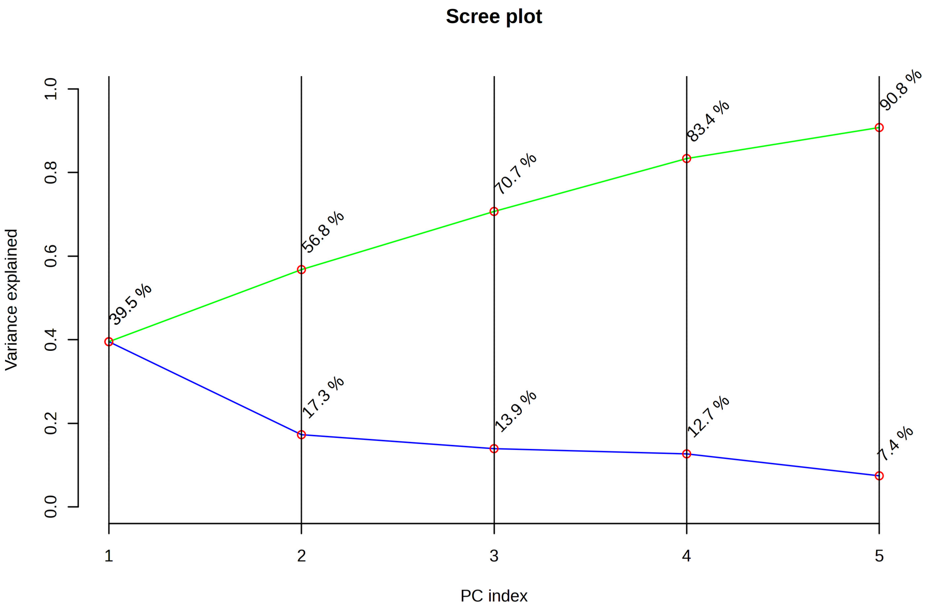

Supplementary Figure S2: Full Hierarchical Clustering Heatmap of All 10 Fruit Samples.

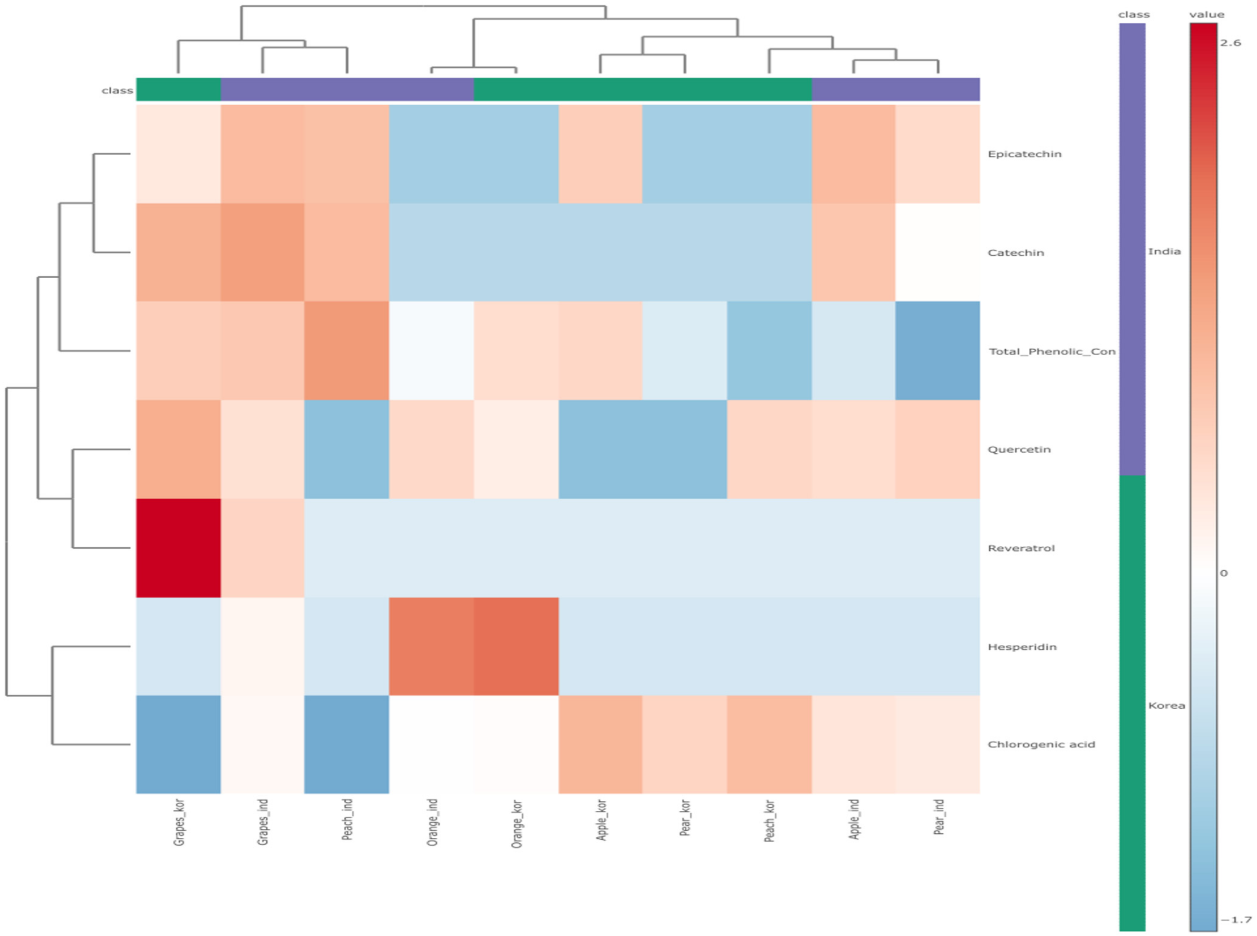

class
Epicatechin
Catechin
Total_Phenolic_Con
Quercetin
Reveratrol
Hesperidin
Chlorogenic acid
Grapes_kor
Grapes_ind
Peach_ind
Orange_ind
Orange_kor
Apple_kor
Pear_kor
Peach_kor
Apple_ind
Pear_ind
class
India
Korea
value
2.6
0
−1.7

Supplementary Figure S3: (A) Docking visualization for 3A1F

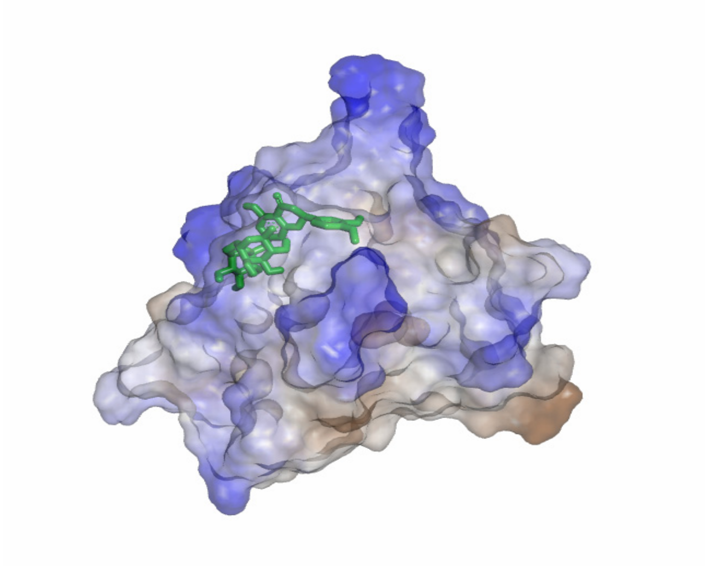

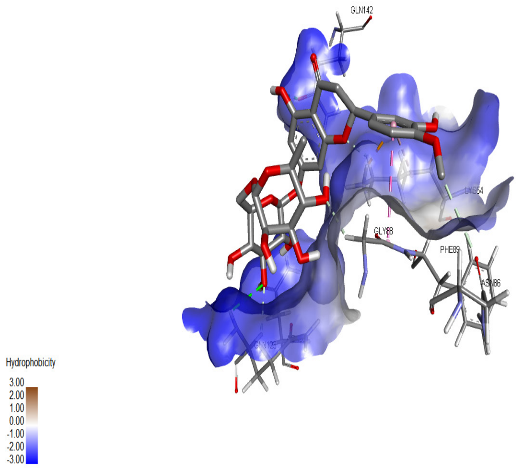

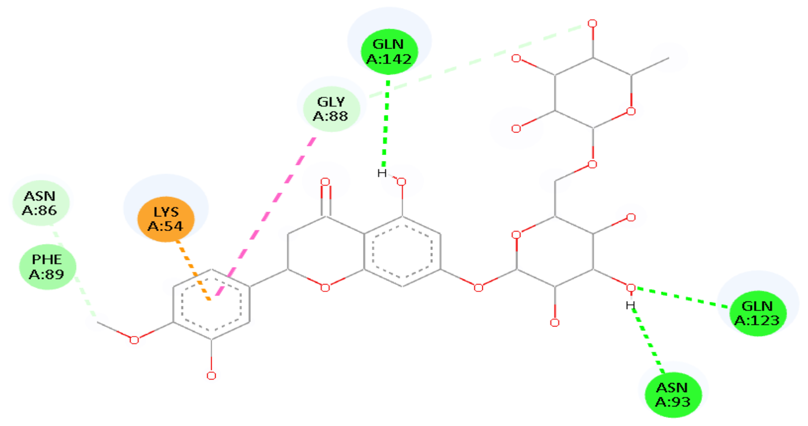

Interactions

- van der Waals
- Conventional Hydrogen Bond
- Carbon Hydrogen Bond
- Pi-Cation
- Amide-Pi Stacked
- Pi-Alkyl

(B) Docking visualization for 7KAD

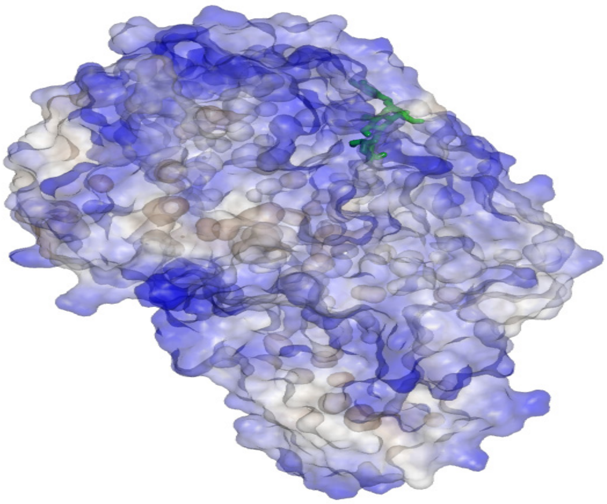

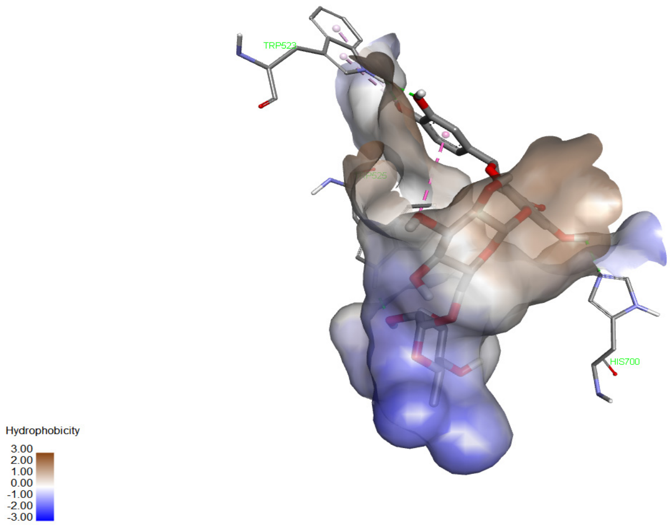

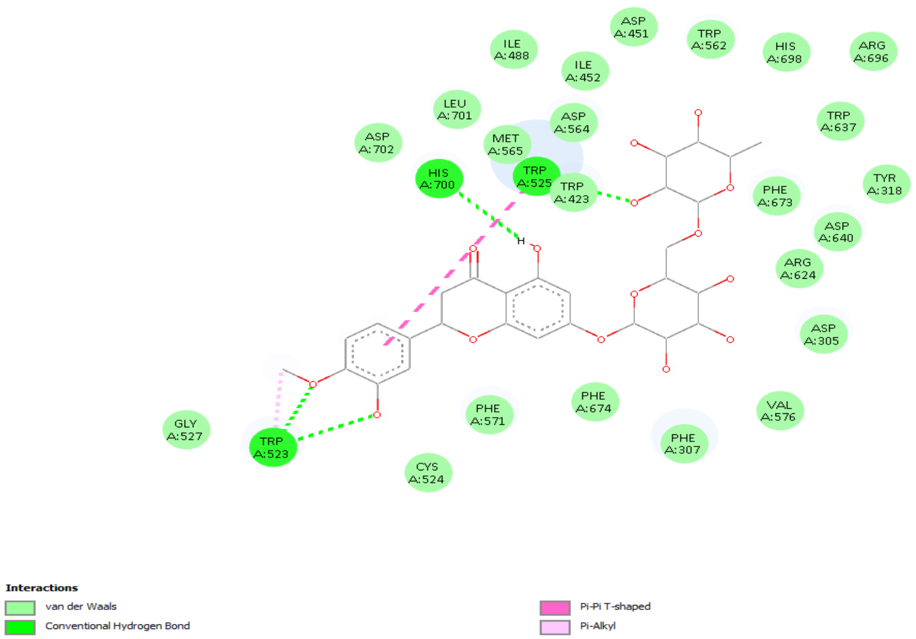
ASP A:451
TRP A:562
HIS A:698
ARG A:696
ILE A:488
ILE A:452
LEU A:701
ASP A:564
TRP A:637
ASP A:702
MET A:565
HIS A:700
TRP A:525
TRP A:423
PHE A:673
TYR A:318
ASP A:640
ARG A:624
ASP A:305
GLY A:527
TRP A:523
PHE A:571
PHE A:674
VAL A:576
PHE A:307
CYS A:524
Interactions
van der Waals
Conventional Hydrogen Bond
Pi-Pi T-shaped
Pi-Alkyl
